# Luxation traumatique invétérée de la hanche

**DOI:** 10.11604/pamj.2015.22.100.7238

**Published:** 2015-10-06

**Authors:** Abdelillah Rachid, Najib Abdeljaouad, Daoudi Abdelkrim, Yacoubi Hicham

**Affiliations:** 1 CHU Mohammed VI, Faculté de Médecine et de Pharmacie d'Oujda, Oujda, Maroc

**Keywords:** Luxation négligée, prothèse totale de la hanche, nécrose de la tête fémorale, neglected dislocation, total hip prosthesis, necrosis of the femoral head

## Abstract

La luxation traumatique invétérée de la hanche est une affection rare et grave avec une prise en charge difficile et non encore codifiée. L'arthroplastie totale de la hanche reste le traitement chirurgical de choix. Nous rapportons un cas de luxation iliaque invétérée de la hanche chez un jeune patient de 19 ans, traitée par une arthroplastie totale de la hanche. Après un recul de 2 ans, le résultat fonctionnel est satisfaisant, avec une marche indolore sans aide. Le Score de Harris est de 96 et le score de Merle d'aubigné-Postel est de 17.

## Introduction

La luxation traumatique invétérée de la hanche est une affection rare et grave avec une prise en charge difficile et non encore codifiée. Elle est souvent secondaire à un traumatisme violent, où la luxation passe inaperçue devant la gravité des lésions associées. Tout retard de prise en charge aggrave encore le pronostic fonctionnel de la hanche. L’évolution se fait inéluctablement vers la nécrose aseptique de la tête fémorale et l'arthroplastie de la hanche reste l'attitude la plus adoptée. Nous rapportons un cas de luxation invétérée de la hanche, associée à un cal vicieux de la paroi postérieure du cotyle, ayant bénéficié d'une prothèse totale de la hanche.

## Patient et observation

**Figure 1:**
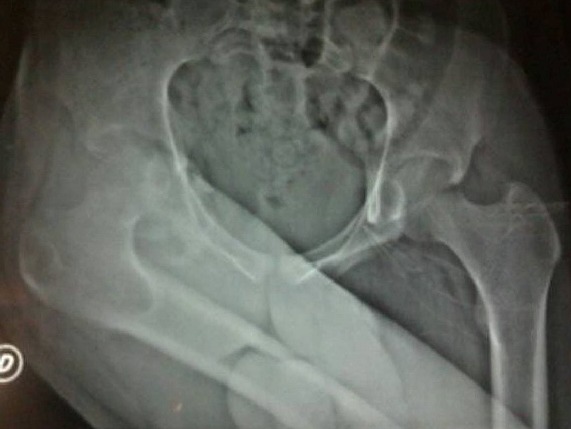
Radiographie standard du bassin montant une luxation négligée de la hanche

**Figure 2:**
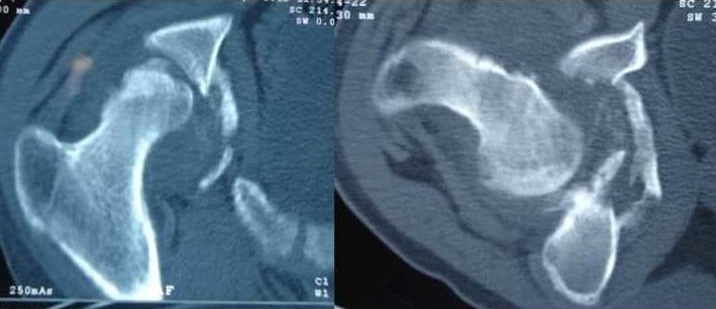
La tomodensitométrie de la hanche droite

**Figure 3:**
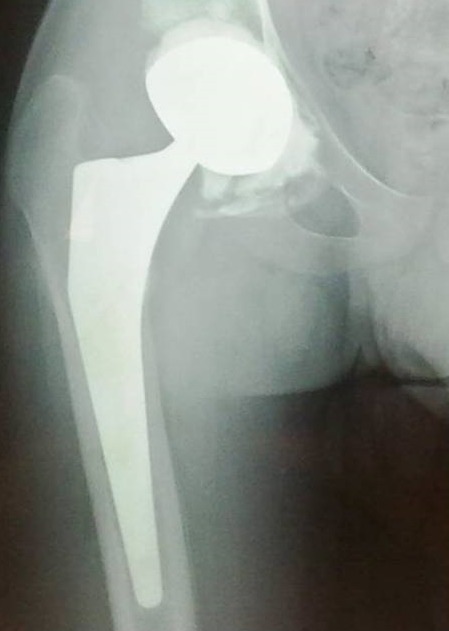
Contrôle radiologique post-opératoire

Mr. Z.M, 19 ans, maçon de profession, sans antécédents pathologiques, victime d'un accident de travail 9 mois auparavant (chute d’échafaudage du 2^ème^ étage), occasionnant chez lui un traumatisme crânien grave pour lequel il a été hospitalisé en milieu de réanimation pendant deux mois. Le patient avait consulté dans notre formation, 9 mois après, pour une attitude vicieuse douloureuse de sa hanche droite depuis le traumatisme. L'examen clinique avait révélé un patient conscient avec une attitude vicieuse irréductible de la hanche droite en adduction, flexion et rotation interne. Il n'y avait pas de paralysie sciatique. La radiographie standard de la hanche droite de face (Figure 1) avait révélé une luxation iliaque négligée de la hanche. La tomodensitométrie de la hanche droite (Figure 2), avait confirmé le diagnostic de luxation avec une nécrose de la tête fémorale et un cal vicieux de la paroi postérieure du cotyle. Le traitement était chirurgical, avec mise en place par voie d'abord postéro-externe de Moore, d'une prothèse totale de la hanche double mobilité. Le couple de frottement utilisé était métal-polyéthylène (Figure 3). Une ténotomie du muscle droit de la cuisse a été réalisée pour corriger un flessum de la hanche en per-opératoire. Les suites post-opératoires étaient simples. Un protocole de rééducation activo-passive de la hanche a été démarré dès le 2^ème^ jour. Avec un recul de 24 mois, le patient a récupéré des amplitudes articulaires normales et indolores de la hanche. La marche se faisant normalement sans aide et sans boiterie. Le score de Harris est de 96 et le score de Merle d'Aubigné-Postel est de 17.

## Discussion

Une luxation de la hanche est dite négligée quand elle dépasse 3 mois sans réduction (1). La pauvreté des publications dans la littérature confirme le caractère rarissime de cette entité traumatologique. C'est l'apanage du sujet jeune et actif lors d'un traumatisme violant, où la luxation passe inaperçue dans un contexte de polytraumatisé grave (2). La méconnaissance de la luxation s'accompagne d'un certain nombre de troubles anatomo-physiologiques, avec une nécrose quasi constante de la tête fémorale (3). La luxation invétérée est responsable aussi d'une altération de la capsule articulaire, et d'une hypotrophie des muscles péri-articulaires essentiellement le moyen glutéal qui est toujours rétracté rendant difficile la réduction peropératoire de la prothèse, obligeant quelque fois au recours à une trochantérotomie (3). La réduction orthopédique dans les fracture-luxation négligée de la hanche n'a presque aucune indication, vue la consolidation de la fracture du cotyle en cal vicieux et l'encombrement de la cavité cotyloïdienne par un tissu fibreux. Seule la chirurgie peut garantir un résultat fonctionnel satisfaisant. L'arthroplastie totale de la hanche a été le traitement de choix dans la majorité des publications (4). L'arthrodèse de la hanche ou la simple réduction à foyer ouvert ont été rarement utilisées.

L'existence de remaniements anatomo-pathologiques de la tête fémorale et des muscles péri-articulaires rend l'abord chirurgical et les manœuvres réductionnelles difficiles, nécessitant des ténotomies des muscles rétractés à la demande (ischio-jambiers et droit antérieur). La rétraction du moyen glutéal nécessite dans quelques réductions difficiles le recours à une trochantérotomie systématique ou à une ostéotomie de raccourcissement fémorale par analogie au traitement chirurgical des luxations congénitales de la hanche (5). L'analyse des données de la littérature appui encore plus notre conduite thérapeutique, par la mise en place d'une prothèse totale de hanche d'emblée (5). Le recours à une reconstruction du cotyle peut être nécessaire en cas de paroi postérieure déficiente, Hansen (6) a utilisé un greffon cortico-spongieux pour reconstruire le cotyle. Dans notre cas la fracture du cotyle n’était pas très déplacée, sans effet sur la congruence de la cavité cotyloïde. L'atteinte du nerf sciatique varie entre 0 et 10% selon les séries (3). Le risque d'une lésion iatrogène en peropératoire est plus important dans ces affections, expliqué par les modifications des repères anatomiques chez ces patients.

## Conclusion

La luxation traumatique négligée de la hanche est une lésion grave, la prévention passe par un diagnostic précoce, et l'arthroplastie constitue le traitement de choix pour réintégrer le plutôt possible le malade dans la vie sociale.
